# Supplementary material for: SEL1L-HRD1 ER-associated degradation facilitates prohormone convertase 2 maturation and glucagon production in islet α cells
Source: Nat Commun. 2026 Feb 25;17:3202. doi: 10.1038/s41467-026-69928-6 (PMC13057016; doi:10.1038/s41467-026-69928-6)
Supplement: Supplementary file 1 — Supplementary Information [file 41467_2026_69928_MOESM1_ESM.pdf]

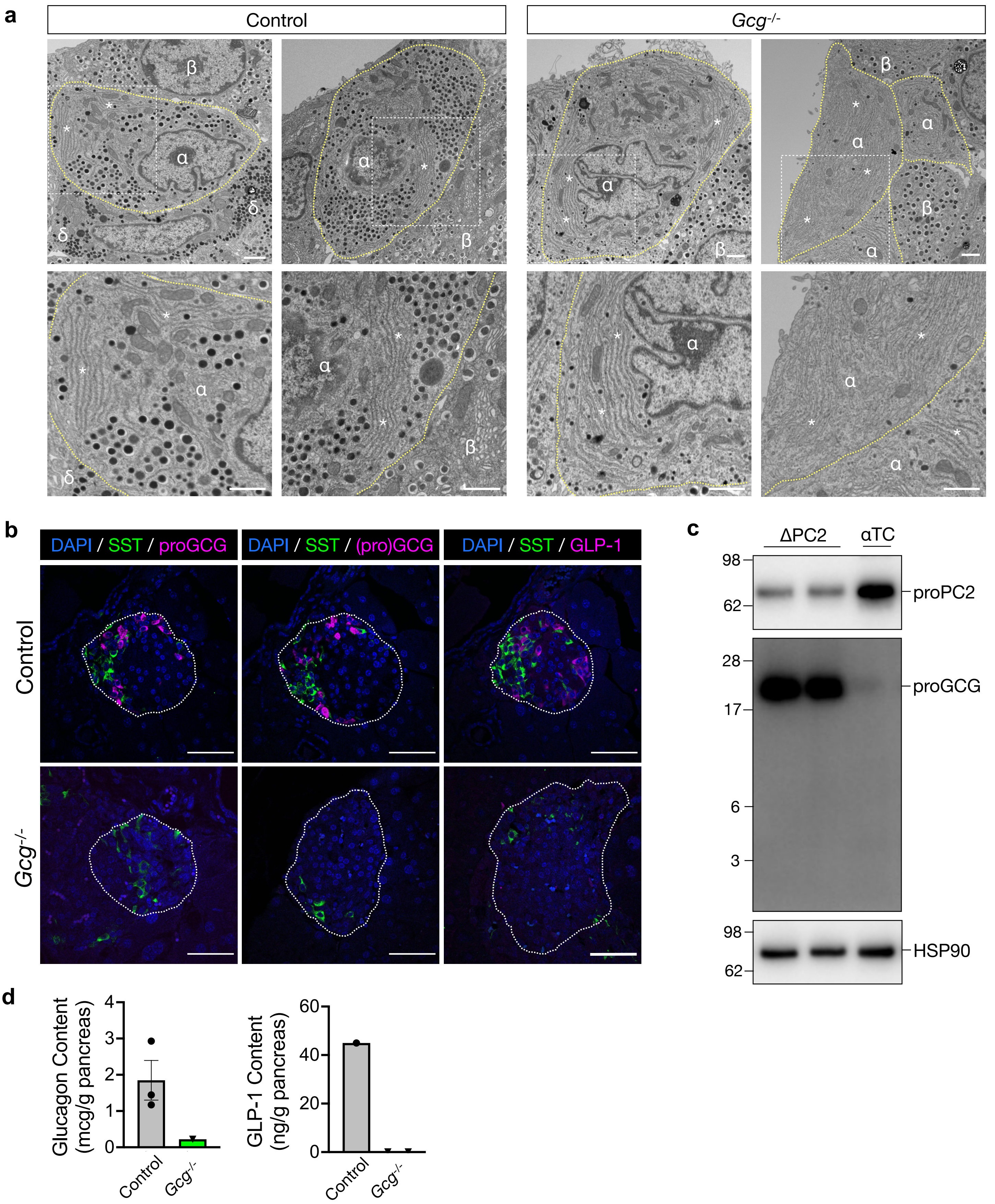

Supplementary Figure 1

Supplementary Figure 1. The endoplasmic reticulum (ER) is a prominent feature of normal and proliferative α cells. a Rough ER (asterisks) in α cells (yellow dashed lines) from *Gcg*$^{-/-}$ mice and littermate controls. Scale bars, 1 μm. **b** Representative islets from *Gcg*$^{-/-}$ mice and controls, immunolabeled for somatostatin (SST; labeling islet δ cells) and proglucagon (proGCG), glucagon (GCG; BMA Biomedicals), or GLP-1. Islet borders are outlined. Scale bars, 50 μm. **c** Western blot of αTC cells with or without CRISPR-mediated inactivation of PC2 using the BMA Biomedicals T-5037 anti-glucagon antibody, revealing reliable detection of the full-length proglucagon protein but no detectable bands at the expected ~ 3 kDa location for mature glucagon. Note that molecular weight markers are listed in kDa. **d** Measurement of glucagon (left) and total GLP-1 (right) in pancreatic extracts from *Gcg*$^{-/-}$ mice and controls. Each data point represents one mouse. Source data are provided as a Source Data file.

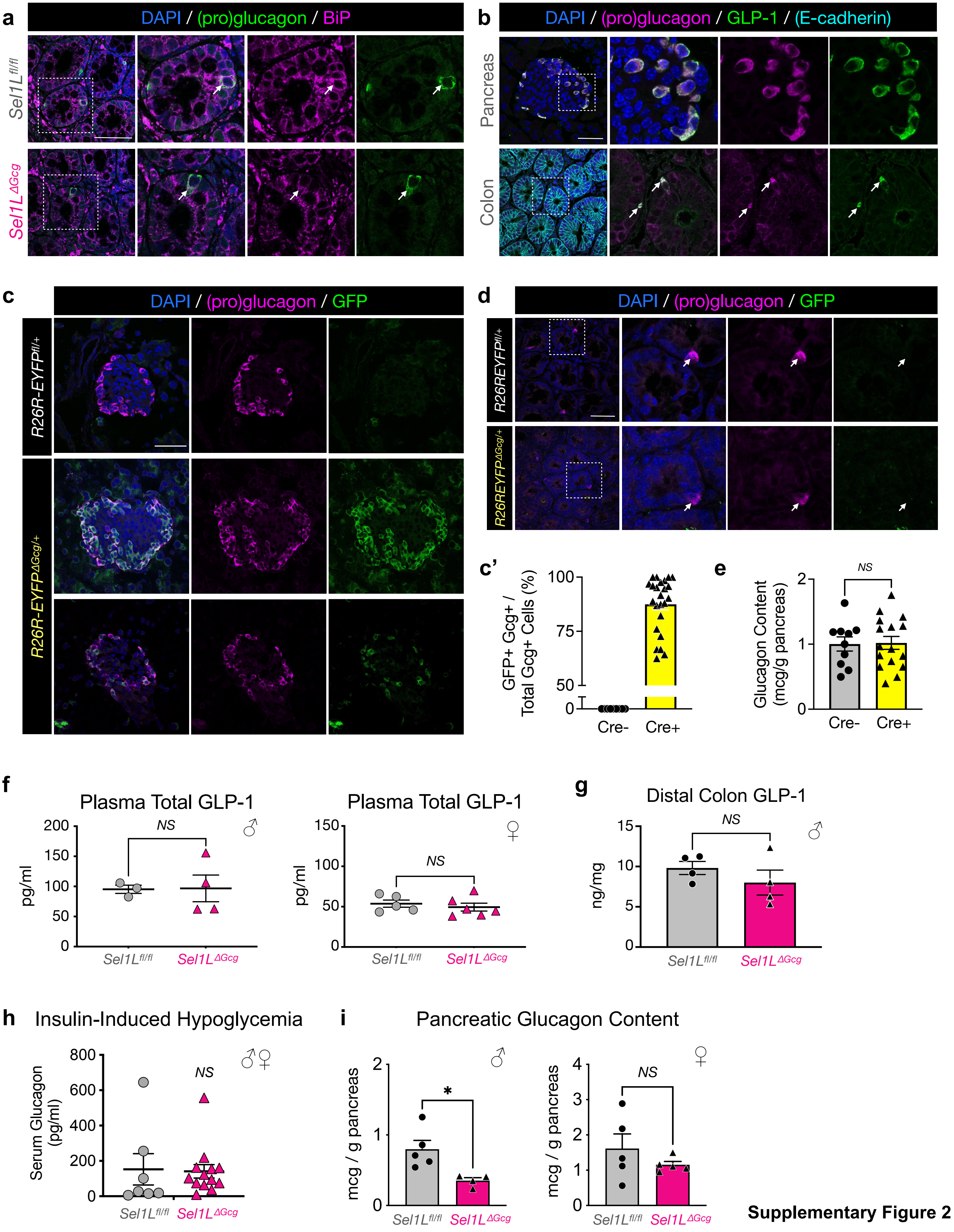

Supplementary Figure 2

Supplementary Figure 2. *Gcg*^{iCre} mice show effective recombination in islet α cells but not intestinal L-cells. a Colon from *Sel1L*$^{\Delta Gcg}$ mice and *Sel1L*$^{fl/fl}$ littermate controls, immunolabeled with antibodies to glucagon (BMA Biomedicals) and BiP. **b** Representative section from pancreas and colon from control mice immunolabeled with antibodies to glucagon (BMA Biomedicals) and GLP-1, along with E-cadherin in the colon sample. Note the similarities in labeling suggest that both antibodies also bind to the full-length proglucagon protein.
c Representative islets from *R26R-EYFP*$^{\Delta Gcg/+}$ (Cre +) mice and *R26R-EYFP*$^{fl/+}$ (Cre -) controls, immunolabeled with antibodies to glucagon (Cell Signaling Technology) and GFP; quantification of GFP+ α cells is shown in **c'** with each data point representing one islet and bars showing mean ± SEM. **d** Intestinal cryosections from *R26R-EYFP*$^{\Delta Gcg/+}$ mice and Cre-negative *R26R-EYFP*$^{fl/+}$ controls, immunolabeled with antibodies to (pro)glucagon (Cell Signaling Technology) and GFP. Scale bars, 50 µm. **e** Pancreatic glucagon content in *R26R-EYFP*$^{\Delta Gcg/+}$ (Cre +) mice and *R26R-EYFP*$^{fl/+}$ (Cre -) controls at 6-13 weeks of age, mixed sexes. **f** Plasma total GLP-1 (1-26, 7-36, and 9-36) levels following glucose administration in male and female *Sel1L*$^{\Delta Gcg}$ mice and *Sel1L*$^{fl/fl}$ controls. **g** Total GLP-1 extracted from distal colon epithelium in male *Sel1L*$^{\Delta Gcg}$ mice and *Sel1L*$^{fl/fl}$ controls. **h** Serum glucagon values measured *in vivo* 30 minutes after insulin-induced hypoglycemia in combined male and female mice at 11 weeks of age. **i** Acid ethanol-extracted pancreatic glucagon content in male and female 11-week-old mice, separated by sex. For e-i, each data point represents one mouse with bars showing mean ± SEM. An unpaired two-tailed Student's t test was performed for each comparison, with **P* = 0.0193 for the male cohort in i, and *P* > 0.05 (*NS*) for all other comparisons. Specific *P* values are available in the source data are provided as a Source Data file.

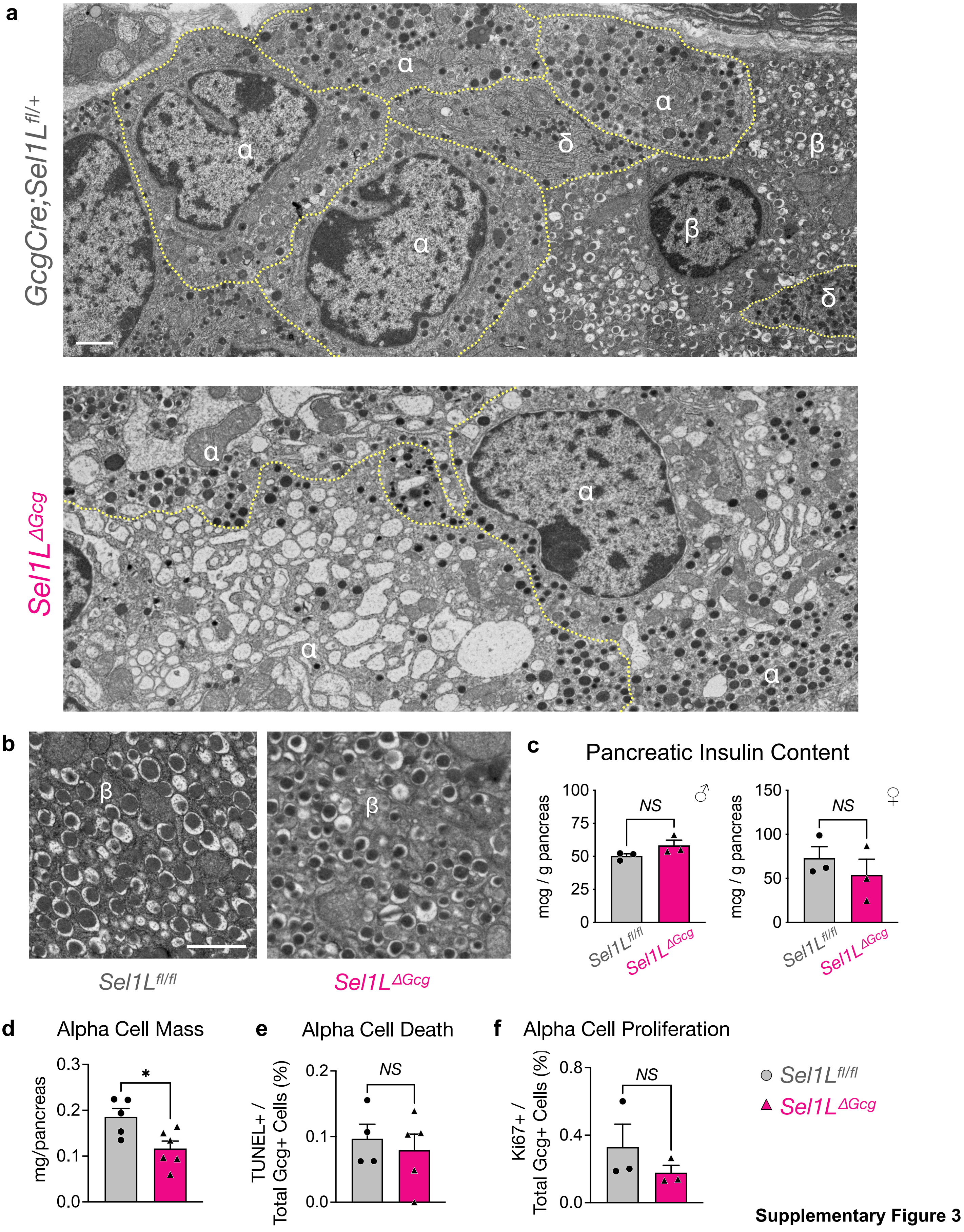

Supplementary Figure 3

Supplementary Figure 3. SEL1L inactivation in pancreatic α cells disrupts ER morphology and glucagon production but does not affect β cell morphology or function. **a** Transmission electron microscopy (TEM) of pancreatic islets from an aged *Sel1L*$^{\Delta Gcg}$ mouse and littermate control. **b** TEM of representative β cells from each genotype. Scale bars in a-b, 1 µm. **c** Acid-alcohol extracted pancreatic insulin content in male and female *Sel1L*$^{\Delta Gcg}$ mice and *Sel1L*$^{fl/fl}$ controls. **d-f** Alpha cell mass (**d**), TUNEL labeling for α cell apoptosis (**e**), and Ki67+ α cell proliferation (**f**) in adult (4-8 month-old) *Sel1L*$^{\Delta Gcg}$ mice (triangles) and *Sel1L*$^{fl/fl}$ controls (circles). In graphs, each point represents data from one mouse, with bars showing mean ± SEM. An unpaired two-tailed Student's t test was performed for each comparison, with *P = 0.0187 in d, and P > 0.05 (*NS*, not significant) for all other comparisons. Specific P values are available in the source data are provided as a Source Data file.

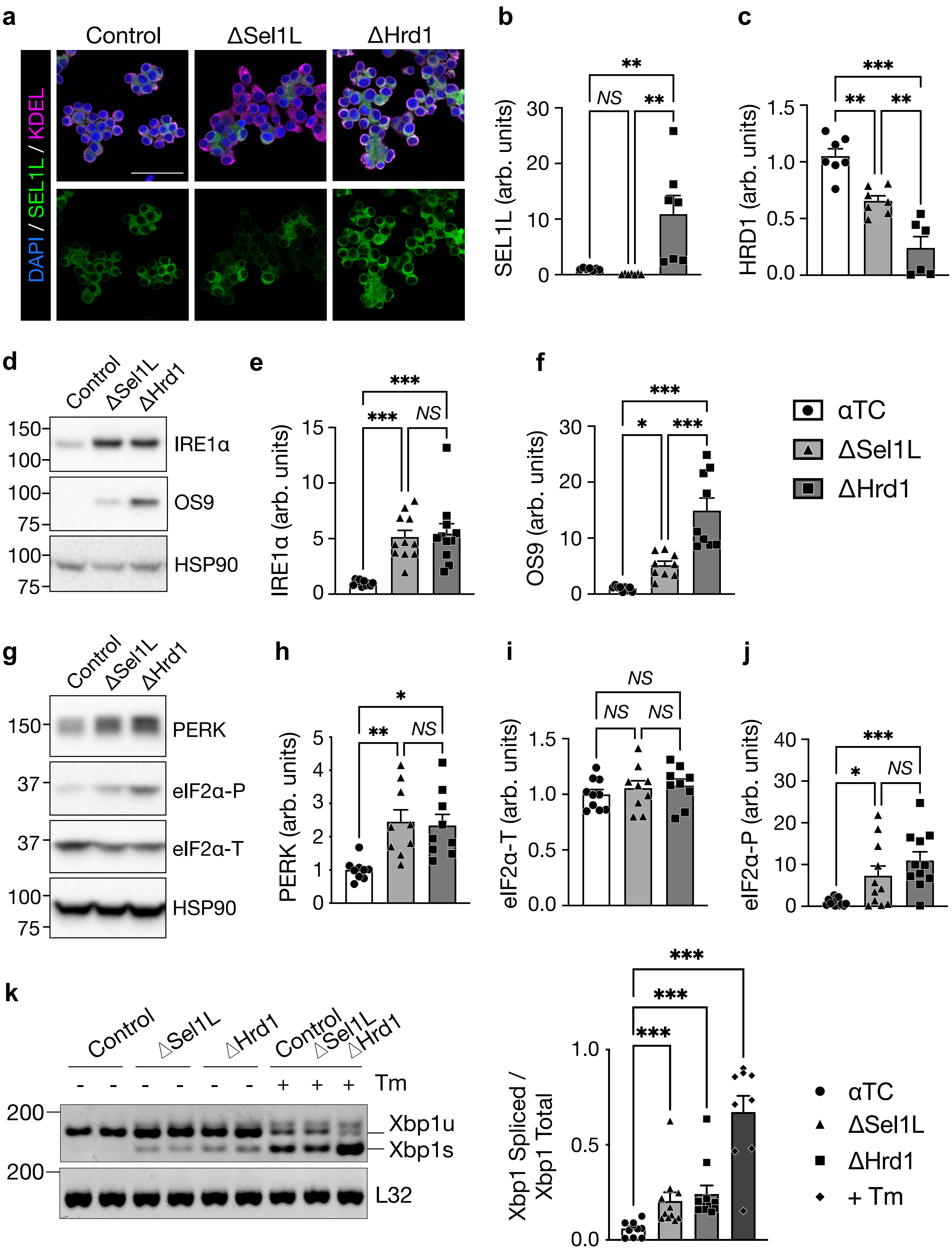

Supplementary Figure 4

Supplementary Figure 4. Inactivation of SEL1L or HRD1 in αTC cells leads to mild activation of the unfolded protein response in vitro. a Immunofluorescence of αTC1-6 cells following CRISPR-mediated deletion of Sel1L (ΔSel1L) or Hrd1 (ΔHrd1) compared to vector-treated controls, labeled for Sel1L and the ER marker KDEL. Note that deletion of Sel1L was incomplete, so Hrd1 deletion was used as an alternative means to inactivate ERAD function. Scale bar, 50 μm. B-C, Quantification of SEL1L (**b**) and HRD1 (**c**) protein expression in αTC1-6 cells following CRISPR-mediated deletion of SEL1L (ΔSel1L, light gray/triangles) or HRD1 (ΔHrd1, dark gray/squares) compared to vector-treated αTC controls (white/circles). Representative Western blots are shown in Fig. 4d. **d-f**, Western blots and quantification of ERAD substrate and UPR sensor IRE1α, and ERAD substrate OS9, in αTC cells with targeted deletion of SEL1L or HRD1. Note that the HSP90 loading control is identical to that shown in Fig. 4d. **g-j** Western blots and quantification of UPR sensor PERK and its downstream effector eIF2α (P, phosphorylated; T, total) in αTC cells with targeted deletion of SEL1L or HRD1. Western blot experiments were performed at least three times with separate biologic replicates, with each data point in graphs representing pooled replicates and summary bars shown as mean ± SEM, expressed as arbitrary units (arb. units). Note that molecular weight markers are listed in kDa in d and g. **k** RT-PCR detection of *Xbp1* mRNA in unspliced (Xbp1u) and spliced (Xbp1s) forms. Quantification was calculated as the ratio of Xbp1s to total Xbp1 (n = 1-3 biologic replicates x 5 separate experiments), with each data point in graph representing pooled replicates and summary bars shown as mean ± SEM. Molecular markers are shown as base pair size. Tunicamycin (Tm) treatment was used as a positive control for activation of IRE1α-mediated Xbp1 splicing; the "+Tm" group is an aggregate of Tm-treated replicates from each genotype. For comparisons, one-way ANOVA with Šidák post-test was performed with *P < 0.05, **P < 0.01, ***P < 0.001, *NS*, not significant (P > 0.05). Specific P values are available in the source data provided as a Source Data file.

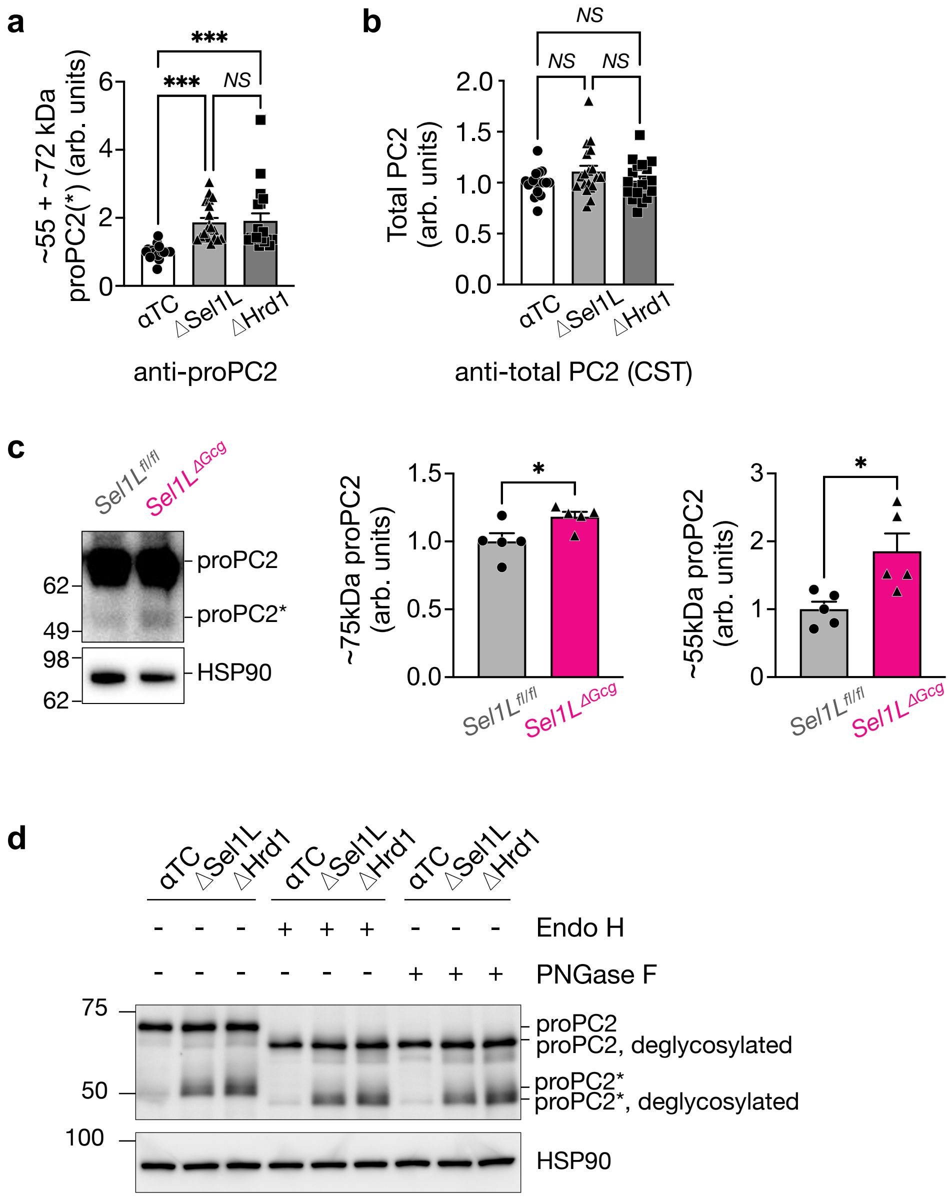

Supplementary Figure 5

Supplementary Figure 5. Additional analysis of (pro)PC2 isoforms in ERAD deficiency.
a-b Quantification of combined proPC2 and proPC2* isoforms (**a**) and all (pro)PC2 isoforms ("Total PC2," **b**) in αTC1-6 cells following CRISPR-mediated deletion of Sel1L (ΔSel1L) or Hrd1 (ΔHrd1) compared to vector-treated αTC controls. Western blot experiments were performed at least three times with separate biologic replicates, with each data point in graphs representing pooled replicates and summary bars shown as mean ± SEM, expressed as arbitrary units (arb. units). ***$P < 0.001$; *NS*, not significant ($P > 0.05$); one-way ANOVA with Šidák post-test.
c Western blot of isolated islet lysates from $Sel1L^{\Delta Gcg}$ mice and $Sel1L^{fl/fl}$ controls, labeled with antibodies to proPC2. Quantification of the protein bands normalized to HSP90 is shown at right. Each data point in the graphs represents an individual biologic replicate of pooled mouse islets, expressed as arbitrary units (arb. units). *$P < 0.05$; unpaired two-tailed Student's t-test.
d Western blot of αTC, ΔSel1L, and ΔHrd1 cells before and after treatment with endoglycosidase H (Endo H) or Peptide:N-glycosidase F (PNGase F). Note that molecular weight markers are listed in kDa in c-d. Specific *P* values are available in the source data provided as a Source Data file.

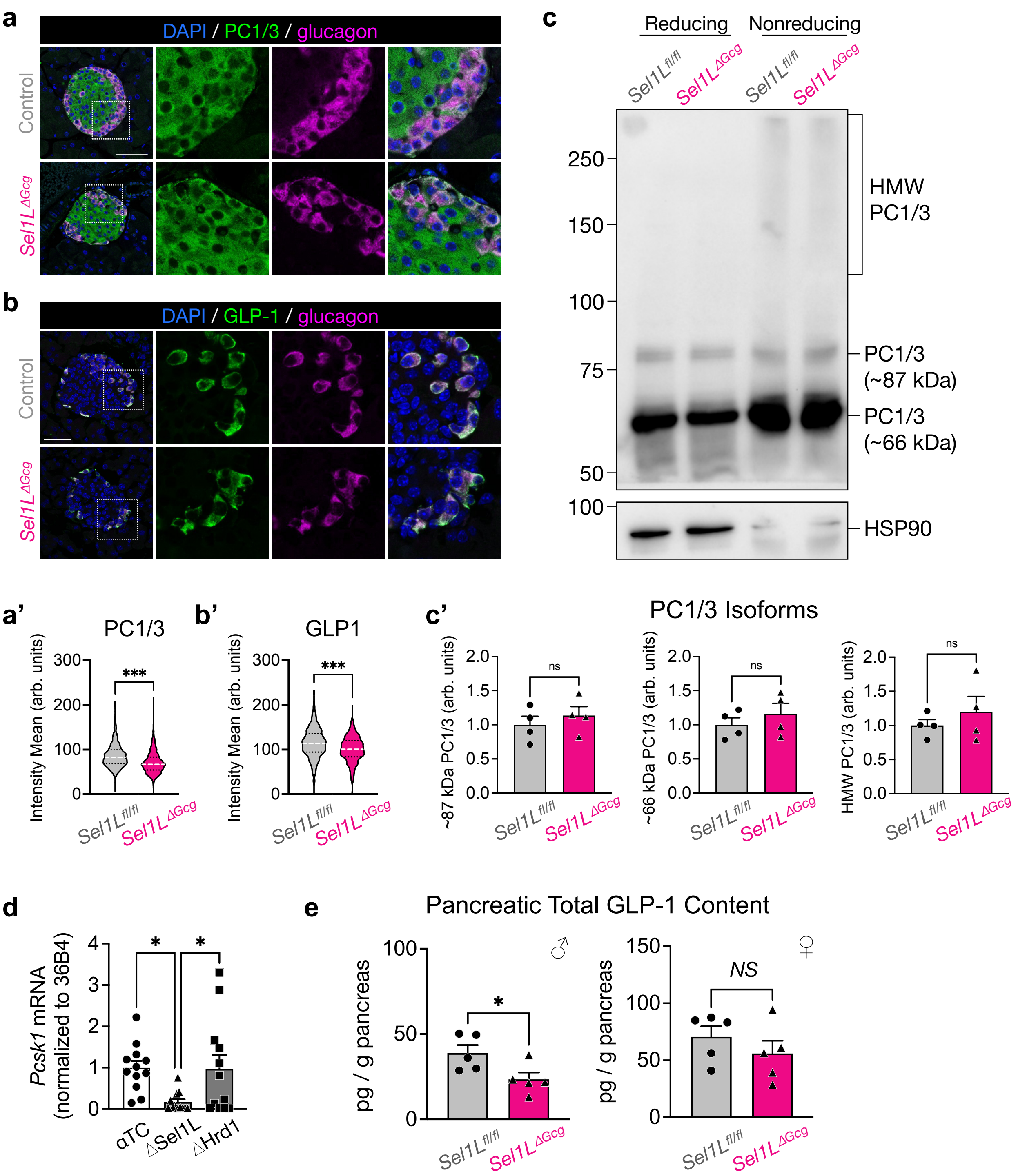

Supplementary Figure 6

Supplementary Figure 6. SEL1L-HRD1 ERAD deficiency has a limited effect on PC1/3 expression and GLP-1 production. a-b Representative islets from *Sel1L*$^{\Delta Gcg}$ mice and *Sel1L*$^{fl/fl}$ controls, immunolabeled for prohormone convertase 1/3 (PC1/3, **a**; RS20 antibody) or glucagon-like peptide 1 (GLP-1, **b**). Scale bars, 25 μm. Violin plots in **a'-b'** show the distribution of immunofluorescence intensity of PC1/3 or GLP-1 in glucagon+ cells, expressed as arbitrary units (arb. units), with median line shown in white and quartile lines in black. In a, 1990 cells were evaluated from two *Sel1L*$^{fl/fl}$ mice and 2029 cells from three *Sel1L*$^{\Delta Gcg}$ mice. In b, 855 cells were evaluated from two *Sel1L*$^{fl/fl}$ mice and 1349 cells from two *Sel1L*$^{\Delta Gcg}$ mice. In a'-b', ***$P < 0.001$, two-tailed Student's t-test. **c** Western blot of islet lysates from *Sel1lL*$^{fl/fl}$ and *Sel1L*$^{\Delta Gcg}$ mice prepared under reducing and nonreducing conditions, labeled with antibodies to PC1/3 (Cell Signaling Technology), with demonstration of high molecular weight (HMW) isoforms. Note that molecular weight markers are listed in kDa. Experiment was performed twice with two biologic replicates of pooled islets. Quantification of each isoform is shown in **c'**, with each data point representing a single replicate, shown as mean ± SEM, expressed as arbitrary units (arb. units). *ns*, not significant ($P > 0.05$); two-tailed Student's t-test.
d Quantitative RT-PCR of the *Pcsk1* gene encoding PC1/3 in αTC cells. Experiment was performed on two separate occasions with six biologic replicates each time. Each data point represents a single replicate, shown as mean ± SEM. *$P < 0.05$; one-way ANOVA with Šidák post-test. **e** Acid ethanol-extracted pancreatic total GLP-1 content in adult (4-8 month-old) mice. Each data point represents data from one mouse, with mean ± SEM shown. *$P < 0.05$, *ns*, not significant ($P > 0.05$); Student's t-test. Specific *P* values are available in the source data provided as a Source Data file.

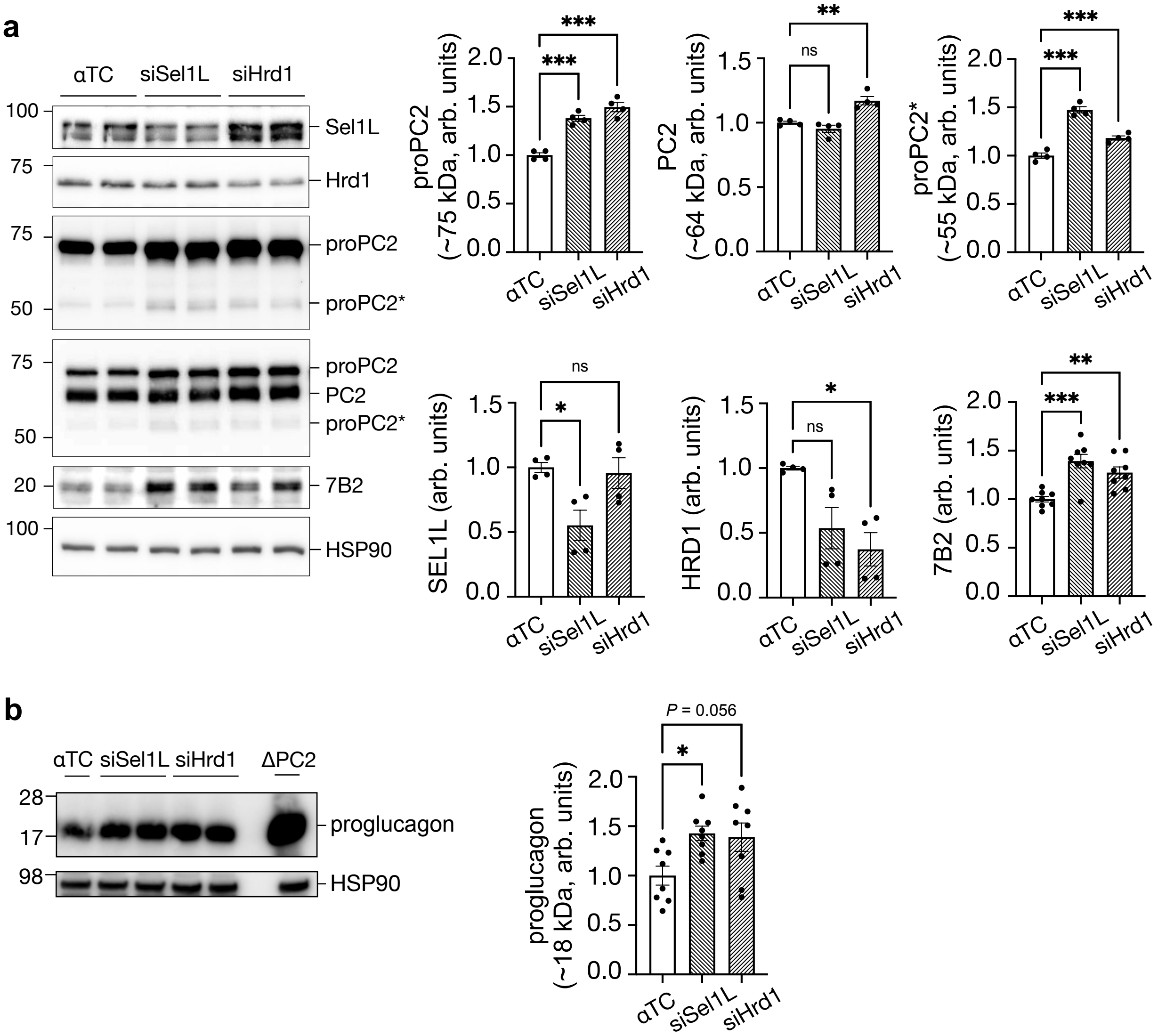

Supplementary Figure 7

Supplementary Figure 7. Acute disruption of SEL1L-HRD1 ERAD disrupts proPC2 maturation and proglucagon processing. Control αTC cells were subjected to siRNA-mediated inactivation of Sel1L or Hrd1 (siSel1L or siHrd1, respectively) and labeled for proteins as shown. **a** Representative Western blots and quantification of the ERAD proteins SEL1L and HRD1, proPC2 isoforms (top middle), total PC2 isoforms (bottom middle), and the proPC2 chaperone 7B2. Experiment was performed twice with two biologic replicates each time.
b siRNA-treated cells were evaluated for expression of proglucagon-derived peptides using the ABclonal anti-glucagon antibody. As a control, note the lack of proglucagon-to-glucagon processing in αTC cells with CRISPR-mediated inactivation of PC2 (ΔPC2). Experiment was performed three times, with 2-4 biologic replicates each. In a-b, each protein band was normalized to the HSP90 loading control, and each data point in the graphs represents an individual replicate, expressed as arbitrary units (arb. units). Note that molecular weight markers are listed in kDa. *$P < 0.05$, **$P < 0.01$, ***$P < 0.001$; *NS*, not significant ($P > 0.05$); one-way ANOVA with Šidák post-test. Specific *P* values are available in the source data provided as a Source Data file.

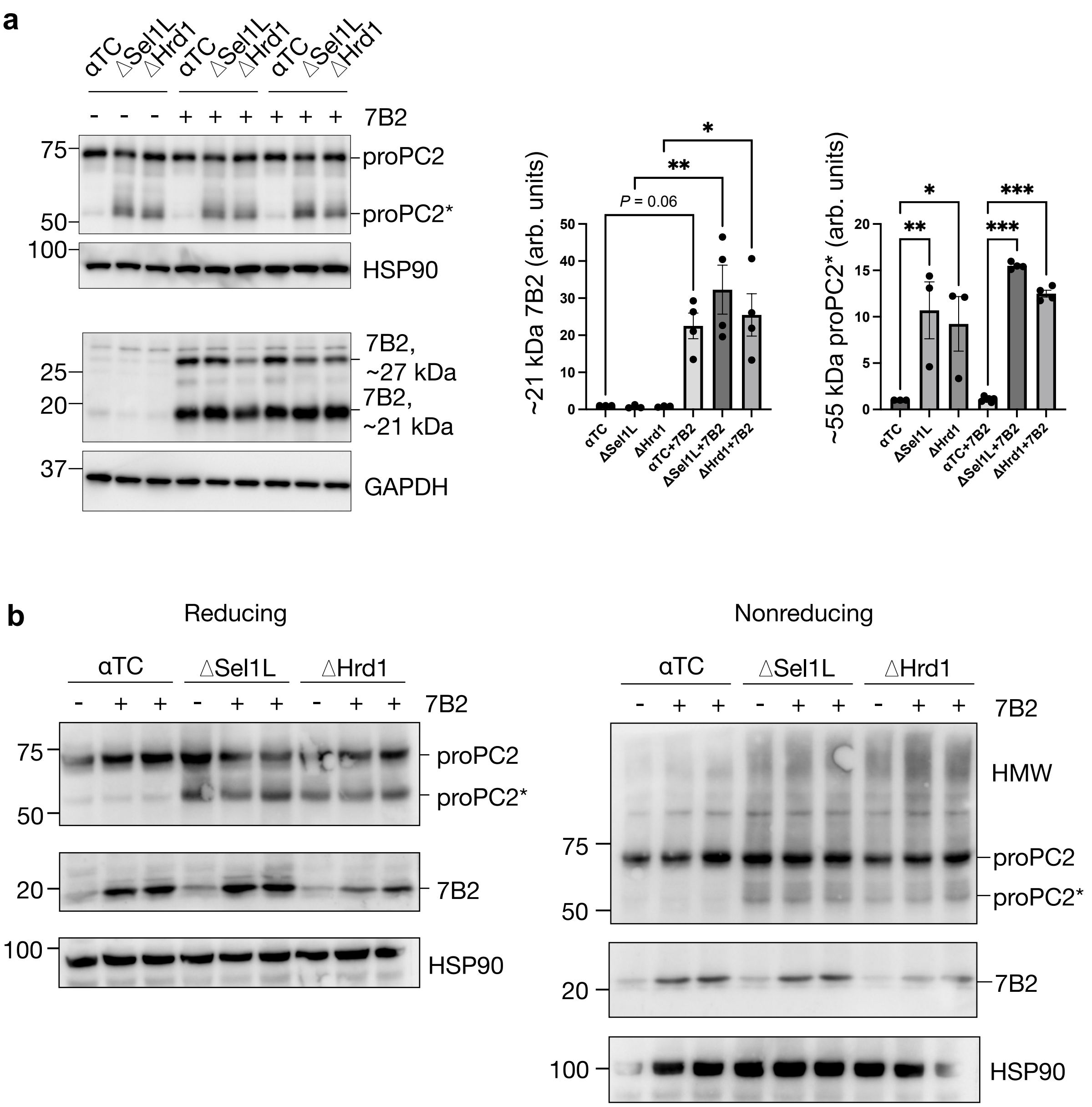
a
αTC ΔSel1L ΔHrd1 αTC ΔSel1L ΔHrd1 αTC ΔSel1L ΔHrd1
7B2
75
50
proPC2
proPC2*
100
HSP90
25
20
7B2, ~27 kDa
7B2, ~21 kDa
37
GAPDH
~21 kDa 7B2 (arb. units)
P = 0.06
**
*
αTC
ΔSel1L
ΔHrd1
αTC+7B2
ΔSel1L+7B2
ΔHrd1+7B2
~55 kDa proPC2* (arb. units)
***
b
Reducing
Nonreducing
HMW
7B2
HSP90

Supplementary Figure 8. Overexpression of 7B2 cannot rescue proPC2* aggregation in ERAD-deficient cells. a Representative Western blots and quantification of proPC2* (top) and 7B2 (bottom) in control and CRISPR-treated ΔSel1L and ΔHrd1 cells that were transfected with a plasmid expressing rat 7B2. **b** Cells without and with 7B2 overexpression were evaluated under reducing and non-reducing conditions to evaluate for high molecular weight (HMW) forms of proPC2(*). Note that molecular weight markers are listed in kDa. Experiments were performed at least twice, with 1-2 biologic replicates each time. In a, each protein band was normalized to HSP90 or GAPDH loading controls. Each data point in the graphs represents an individual replicate, expressed as arbitrary units (arb. units). *$P < 0.05$, **$P < 0.01$, ***$P < 0.001$; one-way ANOVA with Šidák post-test. Note only the most relevant statistical comparisons are shown for clarity. Specific *P* values are available in the source data provided as a Source Data file.

SUPPLEMENTARY TABLES

Supplementary Table 1. Summary of key antibodies used. WB, Western blot; IHC(-P), immunohistochemistry (-paraffin); IF, immunofluorescence; ELISA, enzyme-linked immunosorbent assay; IP, immunoprecipitation.

Antibody Target	Source	Antigen	Known Species Reactivity (Tested Applications)	Host Species	Reference(s)
Glucagon	Peninsula Labs/ BMA Biomedicals T-5037	Synthetic peptide H-His-Ser-Gln-Gly-Thr-Phe-Thr-Ser-Asp-Tyr-Ser-Lys-Tyr-Leu-Asp-Ser-Arg-Arg-Ala-Gln-Asp-Phe-Val-Gln-Trp-Leu-Met-Asn-Thr-OH coupled to carrier protein	Human, mouse, porcine, rat (IHC)	Guinea pig	[91]
Glucagon	Cell Signaling Technology 2760S	Synthetic peptide corresponding to residues surrounding Tyr62 of human glucagon protein	Human, mouse, rat (IHC, IF)	Rabbit	[92]
Glucagon	ABclonal A22702	Synthetic peptide corresponding to residues 21-120 of human proglucagon protein	Human, mouse, rat (WB, IHC-P, IF, ELISA)	Rabbit	No published references available
Proglucagon	Cell Signaling Technology D16G10 XP® 8233	Synthetic peptide corresponding to residues surrounding Ala137 of human proglucagon protein	Human, mouse, rat (IHC, IF)	Rabbit	[93,94]
GLP-1 (7-37)	Peninsula Labs/ BMA Biomedicals T-4363	Synthetic peptide H-Hys-Ala-Glu-Gly-Thr-Phe-Thr-Ser-Asp-Val-Ser-Ser-Tyr-Leu-Glu-Gly-Gln-Ala-Ala-Lys-Glu-Phe-Ile-Ala-Trp-Leu-Val-Lys-Gly-Arg-Gly-OH coupled to carrier protein	Human, bovine, guinea pig, mouse, rat (IHC)	Rabbit	[95]
ProPC2	Homemade	His58 to Asp80 of mouse proPC2 coupled to hemocyanin	Mouse (WB, IF)	Rabbit	[14]
PC2	Cell Signaling Technology D1E1S XP® 14013s	Synthetic peptide corresponding to residues surrounding Pro194 of human PC2 protein	Human, mouse, rat (WB, IP, IHC, IF)	Rabbit	[96]
PC2 (C-terminus)	Homemade	C-terminal YERSLQSILRKN coupled to succinylated hemocyanin	Mouse (WB)	Rabbit	[39,97]
7B2	Homemade	Residues 23-39 of rat 7B2	All vertebrate species (WB)	Rabbit	[61]
PC1/3	Cell Signaling Technology 18030S	Synthetic peptide corresponding to residues surrounding Arg364 of human PC1/3 protein	Human, mouse, rat (WB)	Rabbit	[98]
PC1/3 RS20	Homemade	Mouse prepro-PC3 Cys95-108 + prepro-PC3-(110-122) coupled to hemocyanin	Human, mouse (WB, IHC)	Rabbit	[84-86]

Supplementary Table 2. Characteristics from islet donors.

	**HPAP035 "No DM"**	**ICRH121 "T2DM"**
**Donor age (years)**	35	54
**Donor sex**	Male	Male
**Donor ethnicity**	Caucasian	African-American
**Donor BMI (kg/m^2)**	26.9	33.7
**Donor HbA1c (%)**	5.2	8.0
**Donor diagnosis**	No diabetes	Type 2 diabetes for two years
**Donor cause of death**	Anoxia	Cerebrovascular accident

Supplementary Table 3. CRISPR-targeting sgRNA oligonucleotides

**Gene Name (Protein Name)**	**Forward Sequence (5'→3')**	**Reverse Sequence (5'→3')**
Sel1L (SEL1L)	CACCGGCCAGCAACTACTTTGCCCG	AAACCGGGCAAAGTAGTTGCTGGCC
Syvn1 (HRD1)	CACCGATCCATGCGGCATGTCGGGC	AAACGCCCGACATGCCGCATGGATC
Pcsk2 (PC2)	CACCGACCAGTCATCTGTGTATCGA	AAACTCGATACACAGATGACTGGTC

Supplementary Table 4. Primer sequences for qPCR.

**Gene Name (Protein Name)**	**Forward Sequence (5'→3')**	**Reverse Sequence (5'→3')**
L32	GAGCAACAAGAAAACCAAGCA	TGCACACAAGCCATCTACTCA
Rplp0 (36B4)	AGATTCGGGATATGCTGTTGGC	TCGGGTCCTAGACCAGTGTTC
Xbp1, spliced	TTACGAGAGAAAACTCATGGGC	GGGTCCAACTTGTCCAGAATGC
Pcsk2	AGAGAGACCCCAGGATAAAGATG	CTTGCCCAGTGTTGAACAGGT

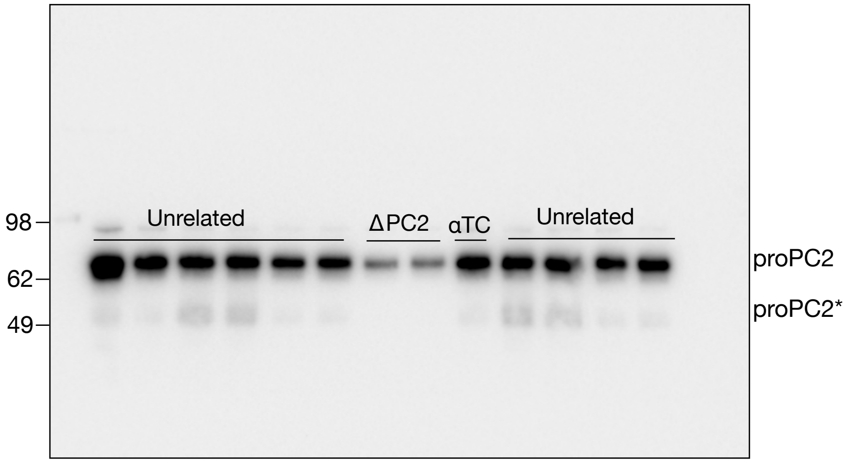

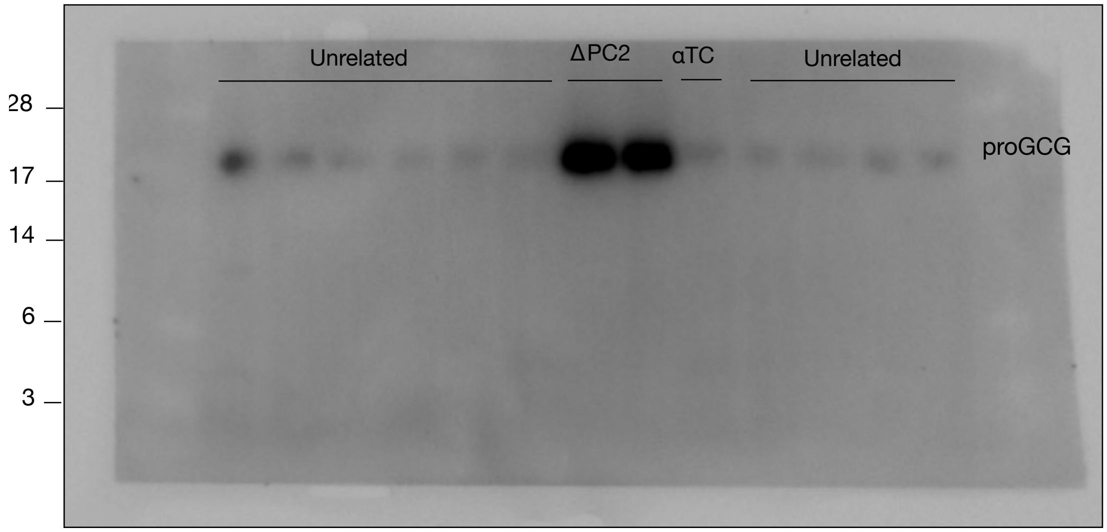

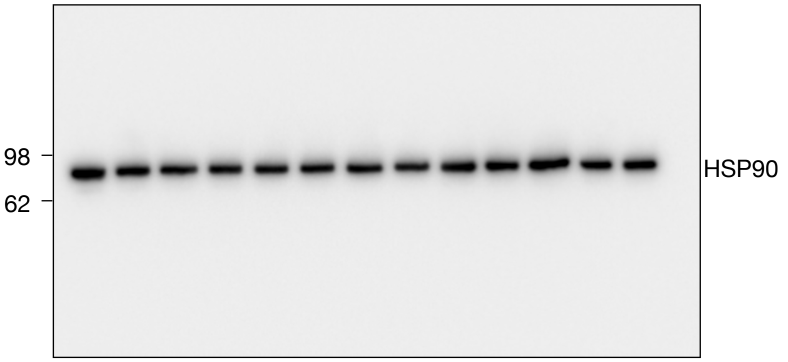

Full blots
Supplementary Figure 1c

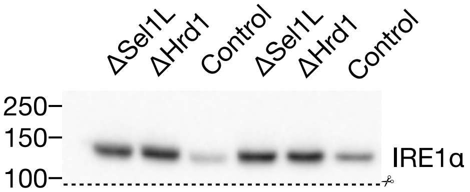

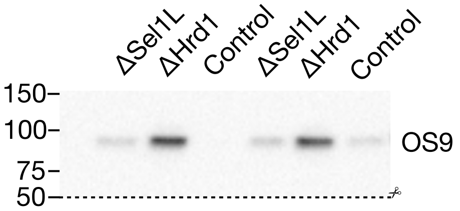

Membranes were physically cut along the dotted line prior to incubation with antibodies

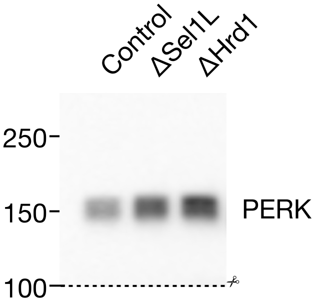

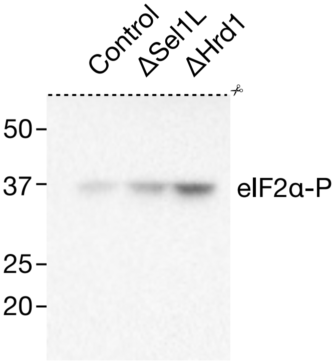

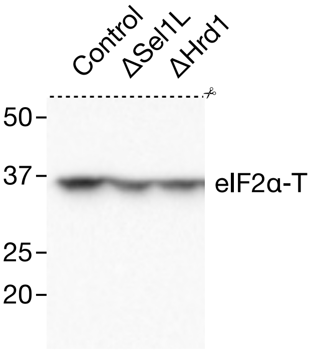

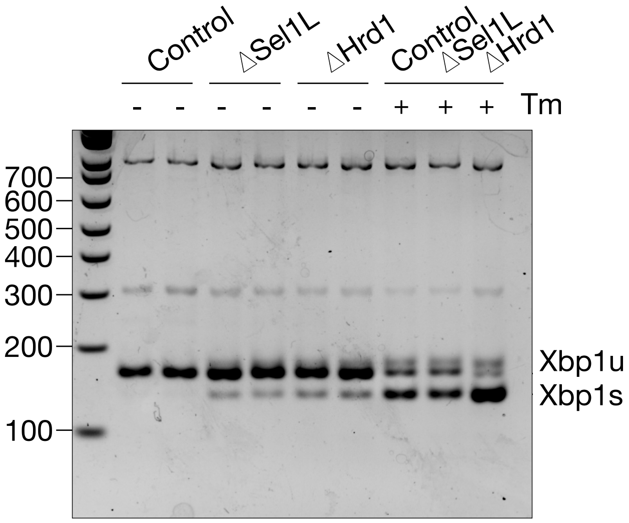

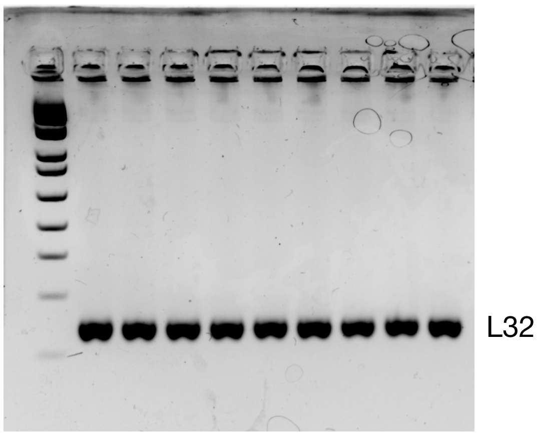

Full blots
Supplementary Figure 4d, g, k

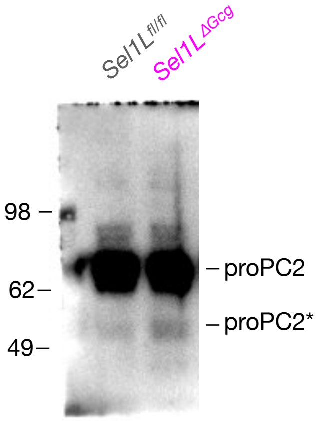

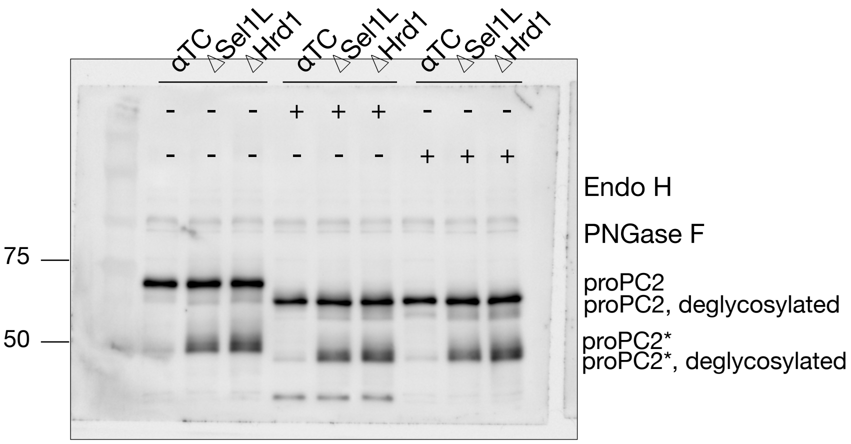

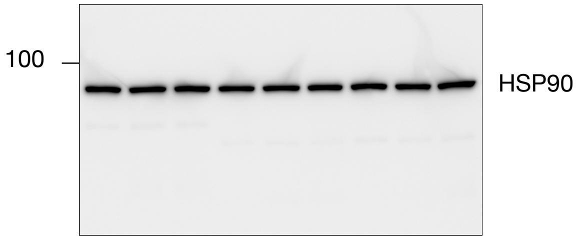

Full blots
Supplementary Figure 5c, d

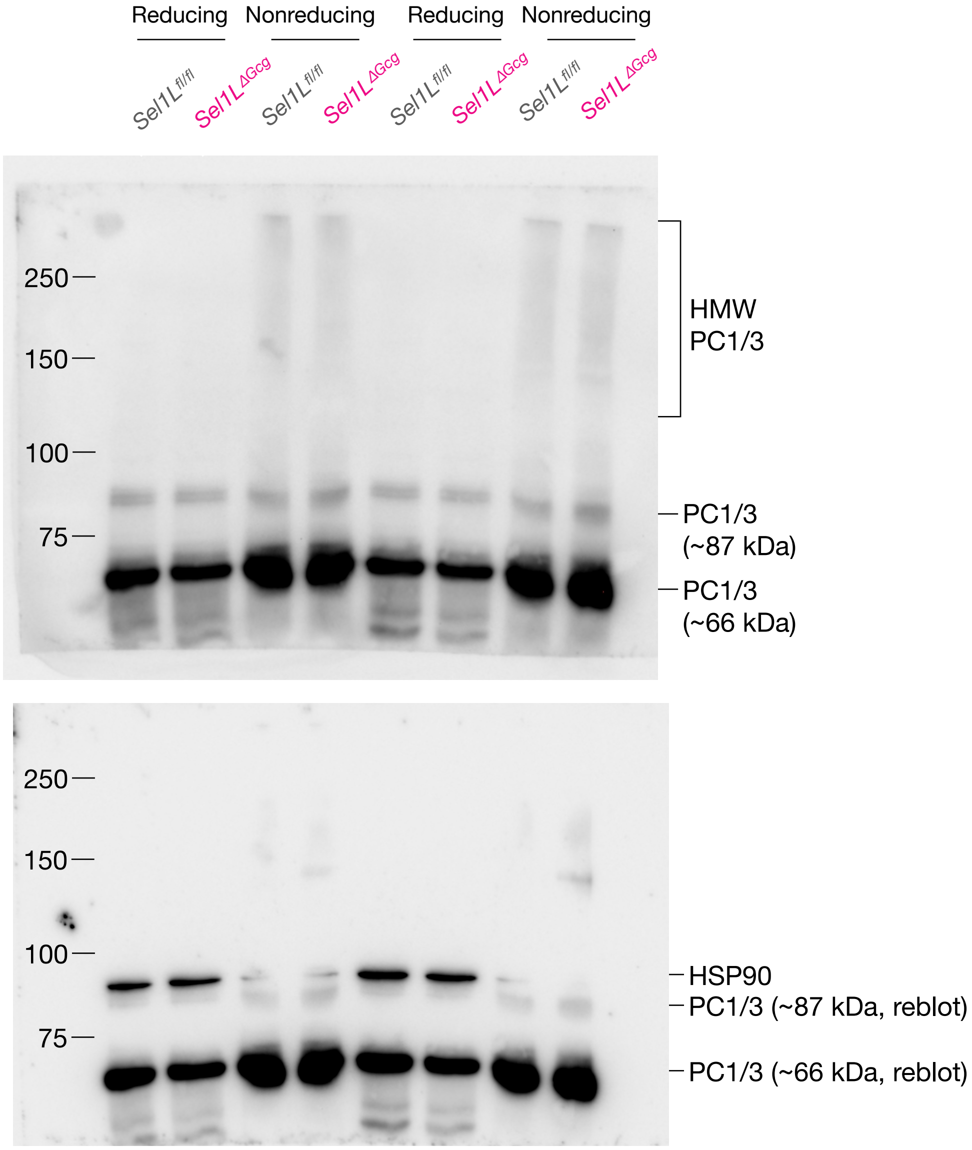

Full blots
Supplementary Figure 6c

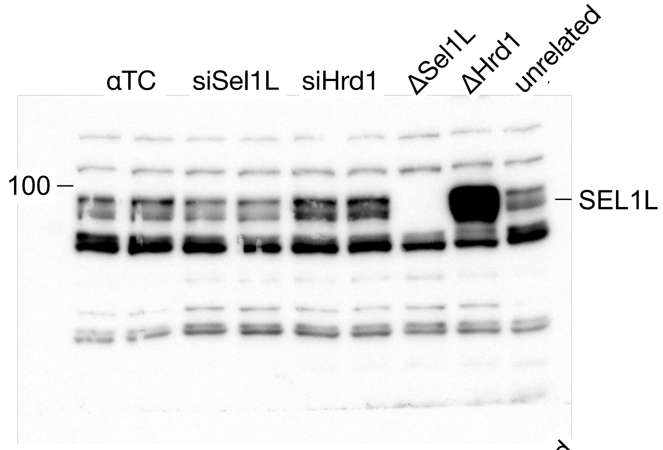

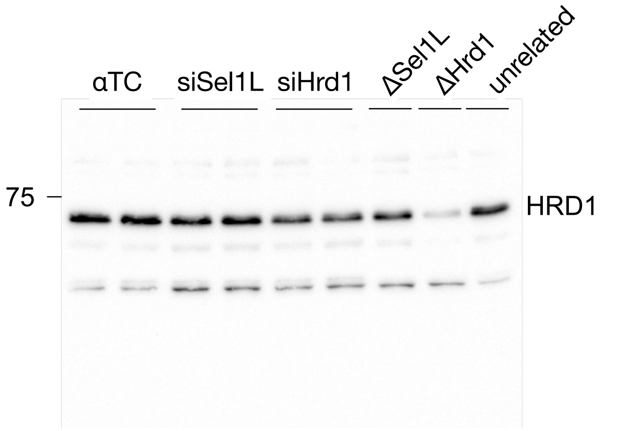

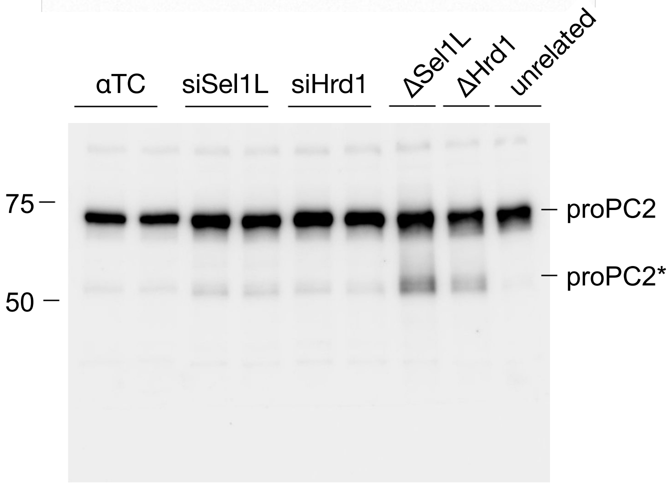

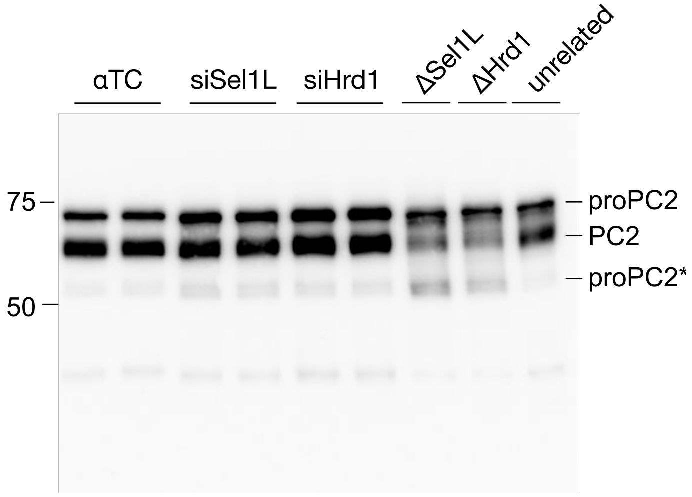

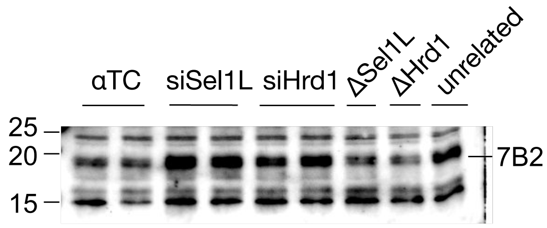

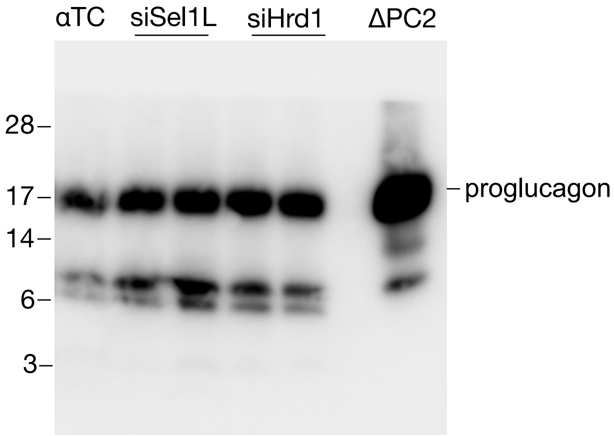

Full blots
Supplementary Figure 7

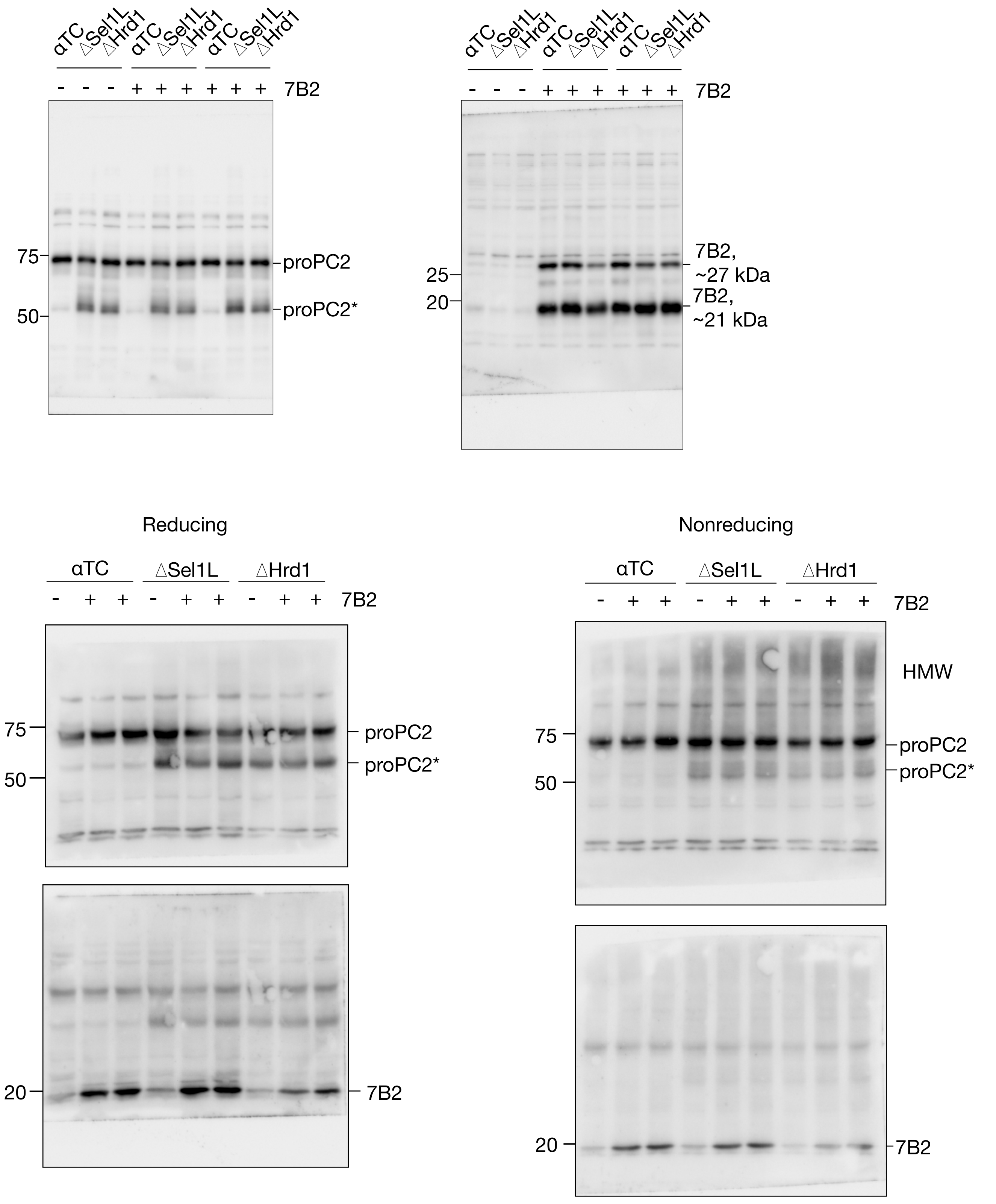

Full blots
Supplementary Figure 8
