# Supplementary material for: SEL1L-HRD1 ER-associated degradation facilitates prohormone convertase 2 maturation and glucagon production in islet α cells
Source: Nat Commun. 2026 Feb 25;17:3202. doi: 10.1038/s41467-026-69928-6 (PMC13057016; doi:10.1038/s41467-026-69928-6)
Supplement: Supplementary file 2 — Reporting summary [file 41467_2026_69928_MOESM2_ESM.pdf]

nature portfolio

Corresponding author(s): Rachel B. Reinert and Ling Qi

Last updated by author(s): Jan 19, 2026

Reporting Summary

Nature Portfolio wishes to improve the reproducibility of the work that we publish. This form provides structure for consistency and transparency in reporting. For further information on Nature Portfolio policies, see our Editorial Policies and the Editorial Policy Checklist.

Statistics

For all statistical analyses, confirm that the following items are present in the figure legend, table legend, main text, or Methods section.

n/a	Confirmed	
☐	☒	The exact sample size (*n*) for each experimental group/condition, given as a discrete number and unit of measurement
☐	☒	A statement on whether measurements were taken from distinct samples or whether the same sample was measured repeatedly
☐	☒	The statistical test(s) used AND whether they are one- or two-sided<br>*Only common tests should be described solely by name; describe more complex techniques in the Methods section.*
☒	☐	A description of all covariates tested
☐	☒	A description of any assumptions or corrections, such as tests of normality and adjustment for multiple comparisons
☐	☒	A full description of the statistical parameters including central tendency (e.g. means) or other basic estimates (e.g. regression coefficient) AND variation (e.g. standard deviation) or associated estimates of uncertainty (e.g. confidence intervals)
☐	☒	For null hypothesis testing, the test statistic (e.g. *F*, *t*, *r*) with confidence intervals, effect sizes, degrees of freedom and *P* value noted<br>*Give P values as exact values whenever suitable.*
☒	☐	For Bayesian analysis, information on the choice of priors and Markov chain Monte Carlo settings
☒	☐	For hierarchical and complex designs, identification of the appropriate level for tests and full reporting of outcomes
☒	☐	Estimates of effect sizes (e.g. Cohen's *d*, Pearson's *r*), indicating how they were calculated

Our web collection on statistics for biologists contains articles on many of the points above.

Software and code

Policy information about availability of computer code

Data collection	Immunofluorescence images were collected with a Nikon A1 or Leica STELLARIS 8 FALCON confocal microscope.<br>proPC2 structure was predicted using AlphaFold2 (https://alphafold.ebi.ac.uk/).
Data analysis	Statistical tests were performed in Graph Pad Prism version 10.0 (Graph Pad Software).<br>Immunofluorescence images were analyzed using the Fiji 2.140 software (Image J) or Imaris software (v10.0, Oxford Instruments).<br>Western blots were imaged with a Bio-Rad ChemiDoc and protein band intensity was quantified using Image Lab software (Bio-Rad).<br>The target sites for CRISPR/Cas9 were selected using the web program (http://www.e-crisp.org/E-CRISP/designcrispr.html).

For manuscripts utilizing custom algorithms or software that are central to the research but not yet described in published literature, software must be made available to editors and reviewers. We strongly encourage code deposition in a community repository (e.g. GitHub). See the Nature Portfolio guidelines for submitting code & software for further information.

Data

Policy information about availability of data

All manuscripts must include a data availability statement. This statement should provide the following information, where applicable:

- Accession codes, unique identifiers, or web links for publicly available datasets
- A description of any restrictions on data availability
- For clinical datasets or third party data, please ensure that the statement adheres to our policy

The materials and reagents used are either commercially available or available upon request. All other data are available in the main text or in the supplementary information and Source Data Files. Source data are provided with the paper.

Research involving human participants, their data, or biological material

Policy information about studies with human participants or human data. See also policy information about sex, gender (identity/presentation), and sexual orientation and race, ethnicity and racism.

Reporting on sex and gender	Our study included human islets from two male donors, outlined in Supplementary Table 2.
Reporting on race, ethnicity, or other socially relevant groupings	We did not analyze data regarding race or ethnicity, though the latter is reported in Supplementary Table 2.
Population characteristics	Available characteristics are reported in Supplementary Table 2.
Recruitment	This manuscript used human islets acquired from the University of Pennsylvania Islet Transplant Center in collaboration with the Human Pancreas Analysis Program (HPAP-RRID:SCR_016202), a Human Islet Research Network (RRID:SCR_014393) consortium (UC4DK112217), and the Integrated Islet Distribution Program (IIDP) (RRID:SCR_014387) through City of Hope (UC4DK098085), supported by Beckman Research Center grant 10028044 (to A.N.).
Ethics oversight	Isolated islets from cadaveric donors were obtained from the Human Islet Resource Center at the University of Pennsylvania through the Human Pancreas Analysis Program and Integrated Islet Distribution Program (IIDP), following the guidelines of the Clinical Islet Transplantation Consortium protocol (https://www.isletstudy.org).

Note that full information on the approval of the study protocol must also be provided in the manuscript.

Field-specific reporting

Please select the one below that is the best fit for your research. If you are not sure, read the appropriate sections before making your selection.

☒ Life sciences ☐ Behavioural & social sciences ☐ Ecological, evolutionary & environmental sciences

For a reference copy of the document with all sections, see nature.com/documents/nr-reporting-summary-flat.pdf

Life sciences study design

All studies must disclose on these points even when the disclosure is negative.

Sample size	At least three mice were used per group for immunofluorescence analyses. For metabolic tests, at least five mice were used per group. Experiments may include data from animals from independent cohorts. Details on sample sizes are provided in the figure legends.
Data exclusions	No animals or samples were excluded from analysis.
Replication	All experiments were repeated at least twice or performed with independent biologic samples. The details are described in the figure legends and methods. Source data was provided with the paper.
Randomization	Mice were grouped based on the age, genotype, and gender. Cells were grown under the same conditions and randomly allocated into different treatment groups without any bias.
Blinding	When experiments were done by one investigator, blindness was not applied. When experiments were done sequentially by different investigators, investigators were blinded to allocation during experiments and outcome assessment.

Reporting for specific materials, systems and methods

We require information from authors about some types of materials, experimental systems and methods used in many studies. Here, indicate whether each material, system or method listed is relevant to your study. If you are not sure if a list item applies to your research, read the appropriate section before selecting a response.

Materials & experimental systems

n/a	Involved in the study
☐	☒ Antibodies
☐	☒ Eukaryotic cell lines
☒	☐ Palaeontology and archaeology
☐	☒ Animals and other organisms
☒	☐ Clinical data
☒	☐ Dual use research of concern
☒	☐ Plants

Methods

n/a	Involved in the study
☒	☐ ChIP-seq
☒	☐ Flow cytometry
☒	☐ MRI-based neuroimaging

Antibodies

Antibodies used

The following primary antibodies were used for immunofluorescence: SEL1L (homemade, 1:200), insulin (Bio-Rad 5330-0104G, 1:500), glucagon (Peninsula Labs/BMA Biomedicals T-5037, 1:200), proglucagon (Cell Signaling Technology 8233S, 1:200), transthyretin (Invitrogen PA580196, 1:500), somatostatin (Abcam ab30788, 1:200), BiP (Abcam 21685, 1:100), OS9 (Abcam ab109510, 1:100), GFP (Abcam 13970, 1:2000), PC2 (Cell Signaling Technology 14013s, 1:800), proPC2 (homemade, 14, 1:200), GLP-1 (Peninsula Labs/BMA Biomedicals T-4363, 1:200), E-cadherin (BD Biosciences 610181, 1:100), PC1/3 (Cell Signaling 18030S, 1:200).

The following primary antibodies were used for Western blotting: anti-SEL1L (homemade, 1:10,000), anti-HRD1 (Proteintech 13473-1, 1:2,000), anti-OS9 (Abcam ab109510, 1:5,000), anti-BiP (Abcam ab21685, 1:5,000), anti-IRE1α (Cell Signaling Technology [CST] 3294, 1:2,000), anti-PERK (CST 3192, 1:1000), anti-eIF2α (CST 9722, 1:1000), anti-phospho-eIF2α (CST 9721, 1:1000), anti-PC2 (CST 14013s, 1:500), anti-PC2 C-terminus (homemade, 1:200), anti-proPC2 (homemade, 1:200), anti-7B2 (homemade, 1:200), anti-PC1/3 (Cell Signaling 18030S, 1:200), anti-glucagon (ABclonal A22702, 1:5000), anti-ubiquitin (Santa Cruz, sc-8017), and anti-HSP90 (Abcam ab13492; 1:2000).

Validation

Details on antigens for key antibodies along with references for prior validation can be found in Supplementary Table 1.

Homemade anti-SEL1L antibody was validated for Western blot (Zhou et al., Science 2020) in mouse and human. We further validated with ΔSel1L TC cells (Figure 5, Supplementary Figure 4).

Commercial antibodies were validated by the manufacturers:

Anti-HRD1 (Proteintech, 13473-1): validated for Western blot, immunofluorescence and immunoprecipitation in mouse and human (https://www.ptglab.com/products/SYVN 1-Anti body-134 73-1-AP.htm).
Anti-OS9 (Abcam, ab109510): validated for Western blot and immunofluorescence in mouse and human (https://www.abcam.com/os9-Antibody-epr42722-ab109510.html).
Anti-BiP (Abcam ab21685): validated for Western blot and immunofluorescence in mouse and human (https://www.abcam.com/en-us/products/primary-antibodies/grp78-bip-antibody-ab21685)
Anti-IRE1α (Cell Signaling Technology 3294): validated for Western blot in mouse and human (https://www.cellsignal.com/products/primary-antibodies/ire1a-14c10-rabbit-mab/3294)
Anti-PERK (Cell Signaling Technology 3192): validated for Western blot in mouse and human (https://www.cellsignal.com/products/primary-antibodies/perk-c33e10-rabbit-mab/3192)
Anti-eIF2α (Cell Signaling Technology 9722): validated for Western blot in mouse and human (https://www.cellsignal.com/products/primary-antibodies/eif2a-antibody/9722)
Anti-phospho-eIF2α (Cell Signaling Technology 9721): validated for Western blot in mouse and human (https://www.cellsignal.com/products/primary-antibodies/phospho-eif2a-ser51-antibody/9721)
Anti-HSP90 (Abcam ab13492): validated for Western blot in human and mouse (https://www.abcam.com/en-us/products/primary-antibodies/hsp90-antibody-ac88-ab13492).
Anti-GLP-1 (Peninsula Labs/BMA Biomedicals T-4363): validated for immunohistochemistry in mouse and human (https://www.bma.ch/en/antibodies/p/t-4363)
Anti-insulin (Bio-Rad 5330-0104G): validated for immunofluorescence in human and mouse (https://www.bio-rad-antibodies.com/static/datasheets/5330-/pig-porcine-insulin-antibody-5330-0104g.pdf)
Anti-transthyretin (Invitrogen PA580196/PA580197): validated for immunohistochemistry in mouse (https://www.fishersci.com/shop/products/transthyretin-polyclonal-antibody-5/PIPA580197)
Anti-somatostatin (Abcam ab30788): validated to react with mouse and human (https://doc.abcam.com/legacy-unpublished/datasheets/com/datasheet_30788.pdf)
Anti-GFP (Abcam 13970): validated for Western blot and immunofluorescence (https://www.abcam.com/en-us/products/primary-antibodies/gfp-antibody-ab13970)
Anti-E-cadherin (BD Biosciences 610181): validated for Western blot and immunofluorescence in mouse and human (https://www.bdbiosciences.com/en-us/products/reagents/microscopy-imaging-reagents/immunofluorescence-reagents/purified-mouse-anti-e-cadherin.610181)
Anti-ubiquitin (Santa Cruz, sc-8017): validated for Western blot in HEK293T cells (https://www.scbt.com/p/ubiquitin-antibody-p4d1).

Eukaryotic cell lines

Policy information about cell lines and Sex and Gender in Research

Cell line source(s)

αTC(1-6) cells were a kind gift from Ernesto Bernal-Mizrachi and also independently obtained from ATCC (CRL-2934).

Authentication	Commercially obtained antibodies were authenticated. We independently confirmed expression of glucagon in these cells.
Mycoplasma contamination	Cells tested negative for Mycoplasma contamination.
Commonly misidentified lines (See ICLAC register)	The cell line is not listed in the database.

Animals and other research organisms

Policy information about studies involving animals; ARRIVE guidelines recommended for reporting animal research, and Sex and Gender in Research

Laboratory animals	Sel1lfl/fl mice (Sun et al., PNAS 2014) on the C57BL/6J background were crossed with B6;129S-Gcgtm1.1(icre)Gkg/J (GcgiCre) mice (Shiota et al., Diabetologia 2017) to generate mice with SEL1L deletion in proglucagon-expressing cells. GcgiCre mice were also crossed with B6.129X1-Gt(ROSA)26Sortm1(EYFP)Cos/J reporter mice (Jackson Laboratory 006148). Glucagon-STOP-flox (Gcg-/-) mice, containing GFP and a poly(A) "stop" signal between exons 2 and 3 of the Gcg gene, were previously described (Chambers et al., Cell Metab 2017).
Wild animals	This study did not include wild animals.
Reporting on sex	Males and females were used equally in experiments, except where sexually dimorphic results were observed and reported.
Field-collected samples	This study did not include field-collected samples.
Ethics oversight	All animal procedures were approved by and done in accordance with the IACUC at the University of Michigan Medical School (PRO00008989/PRO00010658/PRO00011495).

Note that full information on the approval of the study protocol must also be provided in the manuscript.

Plants

Seed stocks	*Report on the source of all seed stocks or other plant material used. If applicable, state the seed stock centre and catalogue number. If plant specimens were collected from the field, describe the collection location, date and sampling procedures.*
Novel plant genotypes	*Describe the methods by which all novel plant genotypes were produced. This includes those generated by transgenic approaches, gene editing, chemical/radiation-based mutagenesis and hybridization. For transgenic lines, describe the transformation method, the number of independent lines analyzed and the generation upon which experiments were performed. For gene-edited lines, describe the editor used, the endogenous sequence targeted for editing, the targeting guide RNA sequence (if applicable) and how the editor was applied.*
Authentication	*Describe any authentication procedures for each seed stock used or novel genotype generated. Describe any experiments used to assess the effect of a mutation and, where applicable, how potential secondary effects (e.g. second site T-DNA insertions, mosiacism, off-target gene editing) were examined.*
